# Errors in Expression and Practice

**Published:** 1903-03

**Authors:** 


### Editorial.

### ERRORS IN EXPRESSION AND PRACTICE.

Dentistry in its origin naturally absorbed many crude ideas, and these have been crystallized into a nomenclature of varied character, obscure meaning, and, oftentimes, misleading in the brief statement of supposed facts. This has been from time to time taken in hand by societies and committees, and attempts have been made to improve ideas by correcting the language in which they have been expressed. This has only been partially successful, and the generally laudable efforts have practically died with the publishing of the reports. This is to be regretted; but the result should have been expected, for the mental habit of expression is one of the strongest and most persistent in human experience. To expect change rapidly is a vain hope. The absorption must come slowly; but because this is true it does not afford sufficient reason for casting aside this and cognate subjects.

The reader of dental literature must be constantly surprised by statements in physiology, pathology, and kindred subjects that either express great carelessness or absolute lack of knowledge; and notwithstanding the higher standard of dental education these seem to increase rather than diminish.

The writer was impressed with the truth of the foregoing statement in reading a paper written by a prominent worker in the dental ranks, where he made use of the expression, "A certain amount of healing inflammation." Exactly what was meant by this it is difficult to determine. Inflammation is not a healing process, but is "the succession of changes which occur in a living tissue, when it is injured, provided that the injury is not of such degree as at once to destroy its structure and vitality." It is, therefore, but a symptom of a series of pathological phenomena, beginning with hyperæmia, and this continued means stagnation of circulation, migration of leucocytes, and blood-plasma, invasion of micro-organisms, liquefaction of tissue,—in a word, an abscess. When this result is taken into consideration, the error of expression becomes apparent. That healing eventually results is to be expected. The removal of this original cause of the inflammation can, however, lead to a return to conditions approximating normal. This is illustrated in the pathological condition known as alveolar abscess. The use of this term is a wrong use of words, yet it would be a waste of effort to criticise the name. It has become fixed in the nomenclature of the profession; but while this is true, a more misleading term has rarely been conceived or adopted. It is needless to state that the inflammation thus described, although pathologically in intimate relation to the process, has no more connection with that than the cement, both being equally affected through their united investing membranes.

In describing this circumscribed abscess writers frequently express themselves in this manner, "Pus and fluids pass out into the *apical space*." This is evidently the result of thoughtless expression, for no one could be ignorant of the fact that there is no such thing as a normal space surrounding the apices of roots; but while it may be thoughtlessness, and not ignorance, it leads equally to a misconception of the anatomy of the jaw as far as implantation of teeth is concerned.

A term that has strangely come into common use is "pericemental abscess." This is meant to apply to an abscess the origin of which is supposed to have been produced through uric acid irritation. Without attempting to discuss the pathological relations of this supposed abscess, it may be said to exhibit no marked difference from the aforementioned form of abscess. Equally with this it has its origin in inflammation of the pericementum and periosteum. Hence the absurdity of giving this name to a condition of uncertain pathology, but in nowise differing from that described, inasmuch as both originate in the pericementum.

The readers of dental periodicals have been surfeited with discussions on "Immediate Root-Filling." If the advocates of this method are to be believed, it does not matter whether the operator has a case of incipient abscess or simply a hyperæmia. In any event proceed to fill at once and all things will end satisfactorily. Indeed, one writer expressed recently, "That a simple discharge of pus, *which can be stopped,* is no bar to the immediate method." If effort be made to reduce the inflammation and cause a stoppage in the formation of pus, it is certainly *not* an immediate method of filling, for this treatment to be successful requires time. If the writer means that he places a filling in a canal with septic conditions and a primary abscess at the apex of the root, he takes risks that eminently entitle him to a suit for malpractice. What can be thought of this paragraph? "How can it be possible . . . that practitioners continue at this late day to treat putrescent root-canals? and if so, why? Do they not know that immediate root-filling in just such cases has been practised successfully for at least twenty years?" That root-filling can be made immediate requires no argument, but when septic conditions exist either in the canal or in the membrane surrounding the root, to fill immediately admits of no excuse save and except a profound ignorance of the pathology of the subject and the resulting sequellæ.

In the treatment of pericementitis the average dental operator confines himself entirely to counterirritation. Whether this be the proper and only course of action cannot be discussed here, but if limited to this simple effort it should be accomplished with some conception of what is required to produce the best results. The philosophy of this process is rarely, if ever, considered. Will the dentist hesitate to use agents that produce vessication of the tissue? Is it, indeed, taken into consideration? It is surmised that the general opinion is that this is the result most desired. The dentist is not so much to blame for this, as it has been part of medical teaching. The importance of keeping the nerves in full activity has not been considered in this connection, and yet upon the sensory nerves must depend the creation of an artificial hyperæmia, the basic principle upon which this counterirritation is founded. It is fallacy, therefore, to make use of such vesicants as cantharides and mustard unless carefully watched, and it is equally an error to make use of a paralyzer, as the tincture of aconite root, thus benumbing the nerves upon which the local increase of the blood current depends.

The dental profession has had it so frequently ingrained in its mental constitution that serumal calculi are the cause of pyorrhœa alveolaris that it would be more than waste energy to attempt to combat it; yet no greater error has ever been implanted upon the dental mind. Calculus is the secondary product and pus organisms are primarily the active irritants.

The dental world hears much of "blind abscess." What is meant by this it is difficult to determine, as there is no such pathological condition existing. If it is meant that an abscess may possibly exist somewhere without an outlet,—a fistula,—such may have a place in general pathology, but not in dental practice. A deep-seated abscess requiring surgical attention might be so classified. An abscess means the formation of pus, and the tendency and function of pus, if it have a function, is to find an exit in the direction of least resistance. This blind-abscess term should have no place in dental nomenclature.

In connection with this is the oft-repeated expression of "curing alveolar abscess." That a root involved in all the conditions connected with an abscess can ever be cured except by a surgical operation is another of those impossible ideas fastened upon the dental mind. Abscess means necrosis of a portion of the cementum, and this, which simulates the sequestrum of necrotic bone, is always a focus of irritation and a perpetual source of offence.

As dentistry progresses new fallacies take the place of, or rather are added to, the old, and of these we will probably never see the end; but whether old or new, they tend to lead the young practitioner into troublesome paths of practice.

In this category may be classed the filling of children's teeth with gold, not warranted with our present histological knowledge; and connected with this is the older practice of always filling with gold without regard to the size of the cavity or the time required to complete the work. This is not now regarded as the best practice, and it is time the professional adoration of Varney and Webb had ceased. In their day they were notable examples of honest work and are worthy of our continued remembrance, but their methods are not adapted, as a whole, to the present advanced thought. The world needs to know the best way to save teeth with the least expenditure of time, money, and suffering to the patient.

This article has only glanced at a few of the errors which in the estimation of the writer mar expression and practice. Each writer and speaker can do much to eliminate these by a careful attention to the formation of ideas into words in writing and speaking. No one, least of all the writer, is free from these defects, and hence it becomes a universal duty to help save the dental profession from a seeming appearance of ignorance when such does not in reality exist.

